# Tau protein in Parkinson’s disease: advances in phosphoproteomics and molecular dynamics simulations

**DOI:** 10.3389/fnagi.2026.1841131

**Published:** 2026-09-25

**Authors:** Jessica Ledesma-Aparicio, Luis Alberto Caro-Gómez, Francisco Javier Padilla-Godínez, Omar Hernández-González, Luis O. Soto-Rojas, Juan Pablo Reyes-Grajeda, Oscar Arias-Carrión, Marcela Palomero-Rivero, Brandt Bertrand, Carlos Munoz-Garay, Magdalena Guerra-Crespo

**Affiliations:** 1 Laboratory of Regenerative Medicine, Department of Physiology, Faculty of Medicine, National Autonomous University of Mexico, Mexico City, Mexico; 2 Department of Mathematics and Physics, Western Institute of Technology and Higher Education, San Pedro Tlaquepaque, Jalisco, Mexico; 3 Laboratory of Neuroscience, Aging and Neurodegeneration (LINEN), Medical Degree Program, Faculty of Higher Studies Iztacala, National Autonomous University of Mexico (UNAM), Tlalnepantla, State of Mexico, Mexico; 4 Protein Structure Laboratory, National Institute of Genomic Medicine (INMEGEN), Mexico City, Mexico; 5 Division of Clinical Neurosciences, National Institute of Rehabilitation, Mexico City, Mexico; 6 Tecnológico de Monterrey, School of Medicine and Health Sciences, Mexico City, Mexico; 7 Department of Cognitive Neuroscience, Division of Neurosciences, Institute of Cellular Physiology, National Autonomous University of Mexico, Mexico City, Mexico; 8 Biological Membrane Physics Laboratory, Institute of Physical Sciences, National Autonomous University of Mexico, Cuernavaca, Morelos, Mexico

**Keywords:** molecular dynamics, neurodegeneration, Parkinson’s disease, phosphoproteomics, synucleinopathies, Tau

## Abstract

Tau dysregulation, particularly abnormal phosphorylation, is increasingly implicated in Parkinson’s disease (PD) and in the pathogenic interaction between Tau and α-synuclein. This review proposes an integrated PD-focused workflow connecting phosphoproteomic discovery, structural modeling, computational drug discovery, and experimental validation. Phosphoproteomic analyses of post-mortem brain tissue, biofluids, and patient-derived dopaminergic neuronal models can identify candidate Tau phosphosignatures associated with vulnerable brain regions, α-synuclein pathology, genetic background, or clinical progression. Molecular dynamics simulations can then evaluate how these signatures alter Tau conformation, microtubule binding, aggregation-prone motifs, membrane association, and pathogenic protein interactions. The resulting structural information can guide ensemble docking, chemoinformatics, and artificial intelligence approaches to prioritize conformer-selective ligands and regulatory targets. Candidate biomarkers should subsequently be validated in independent and longitudinal PD cohorts, whereas therapeutic candidates require mechanistic and functional validation in PD-relevant experimental models before clinical translation. This iterative framework may help distinguish mechanistically relevant Tau phosphoconformers from nonspecific phosphorylation changes, support biomarker-based patient stratification, and focus therapeutic development on structurally defined pathological states. Because much of the available structural evidence derives from Alzheimer’s disease and other tauopathies, direct validation in PD remains essential. By linking PD-derived and disease-relevant molecular evidence with atomistic mechanisms and therapeutic prioritization, this workflow may support biomarker-informed strategies aimed at restoring Tau homeostasis and disrupting pathogenic Tau–α-synuclein interactions.

## 1 Introduction

Parkinson’s disease (PD) is characterized by degeneration of dopaminergic neurons in the substantia nigra pars compacta and by α-synuclein-rich Lewy pathology, including Lewy bodies and Lewy neurites (Spillantini et al., 1997; Braak et al., 2003; Poewe et al., 2017; Stern et al., 2012). However, this classical framework does not fully capture the molecular heterogeneity of the disorder. Tau, traditionally associated with Alzheimer’s disease and primary tauopathies, has increasingly been implicated as a contributor to or modifier of PD pathobiology. Evidence includes genetic association at the MAPT locus, Tau-related changes in post-mortem PD and PD-dementia brains, and experimental Tau–α-synuclein interactions (Simón-Sánchez et al., 2009; Wills et al., 2010; Giasson et al., 2003; Guo et al., 2013; Sengupta et al., 2015; Padilla-Godínez et al., 2025). Together, these findings support a mixed-proteinopathy framework for at least a subset of PD, without implying that PD is a primary tauopathy.

Physiologically, Tau stabilizes neuronal microtubules, supports axonal organization and transport, and contributes to synaptic function (Wang and Mandelkow, 2016; Dixit et al., 2008; Kimura et al., 2013). Alternative splicing of MAPT generates six major adult-brain isoforms that differ in N-terminal inserts and microtubule-binding repeats (Goedert et al., 1989a; Buchholz and Zempel, 2024). Pathological multisite phosphorylation can weaken Tau–microtubule association, promote somatodendritic mislocalization, and favor aggregation-prone states (Biernat et al., 1993; Hoover et al., 2010; Alonso et al., 2001). Kinase–phosphatase dysregulation can further favor hyperphosphorylated Tau, although much causal evidence derives from experimental tauopathy models rather than PD (Patrick et al., 1999; Drewes et al., 1997; Sontag et al., 1996; Park et al., 2016; DeVos et al., 2017).

Tau–α-synuclein convergence provides a mechanistic link to PD. Direct Tau–α-synuclein interactions can stimulate Tau phosphorylation and co-aggregation (Jensen et al., 1999; Giasson et al., 2003; Badiola et al., 2011). Models of α-synuclein overexpression also show increased Tau phosphorylation in PD-relevant brain regions (Kawakami et al., 2011; Lu et al., 2020; Frasier et al., 2005; Haggerty et al., 2011; Kaul et al., 2011). Both proteins can propagate between cells in experimental systems (Desplats et al., 2009; Frost et al., 2009; Clavaguera et al., 2009; Luk et al., 2012). Direct Tau–α-synuclein interactions and cross-seeding can further promote aggregation and spreading (Waxman and Giasson, 2011; Sengupta et al., 2015; Pan et al., 2022). Human neuropathological studies associate coexisting Tau and α-synuclein pathologies with a more aggressive clinical course (Irwin et al., 2017). These observations support Tau as a potential modifier of neuronal vulnerability and disease progression rather than a uniform determinant of PD severity.

Clinical biomarker evidence remains heterogeneous. Tau-related abnormalities are most consistently associated with cognitive impairment and progression to dementia, whereas associations with motor progression vary across cohorts (Irwin et al., 2017; Coughlin et al., 2019; Hall et al., 2014; Mollenhauer et al., 2017). Longitudinal studies have linked cerebrospinal-fluid or plasma phosphorylated Tau to motor complications, motor worsening, or dementia conversion (Hall et al., 2014; Lin et al., 2023; Bissacco et al., 2025; Li et al., 2026). However, these signals may partly reflect concomitant Alzheimer-type pathology rather than a PD-specific Tau process (Pagonabarraga et al., 2022; Tropea et al., 2026). Tau biomarkers therefore remain candidates for stratification and progression monitoring rather than established PD-specific markers (Baek et al., 2021; Pilotto et al., 2024; Li et al., 2026).

Recent phosphoproteomic and structural approaches provide an opportunity to resolve this heterogeneity. Tau contains approximately 85 potential phosphorylation sites, many identified experimentally by mass spectrometry (Hanger et al., 2007). Specific phosphorylation patterns have also been associated with aggregation and neuronal dysfunction (Despres et al., 2017; Hoover et al., 2010; DeVos et al., 2017). Molecular dynamics simulations can test how defined PTM patterns alter Tau conformation, microtubule binding, aggregation-prone motifs, membrane association, and protein interactions (Lyons et al., 2014; Man et al., 2023; Castro et al., 2023). However, most high-resolution Tau phosphoproteomic and structural datasets derive from Alzheimer’s disease or other tauopathies, so their relevance to PD requires direct validation.

Accordingly, this review is organized around an integrated PD-focused workflow. Phosphoproteomic analyses of PD tissues, biofluids, and disease-relevant neuronal models can identify reproducible Tau phosphosignatures associated with α-synuclein pathology, genetic background, neuronal vulnerability, or clinical progression (Wills et al., 2010; He et al., 2020; Bogetofte et al., 2023; Rutledge et al., 2024). Molecular dynamics can then test their structural consequences, and the resulting conformational information can guide state-specific docking, chemoinformatics, artificial intelligence, and therapeutic-target prioritization (Amaro et al., 2018; Vamathevan et al., 2019; Dhar et al., 2025). Candidate biomarkers and therapeutic mechanisms must ultimately be validated in PD-relevant experimental systems and independent patient cohorts (Figure 1).

**Figure 1:**
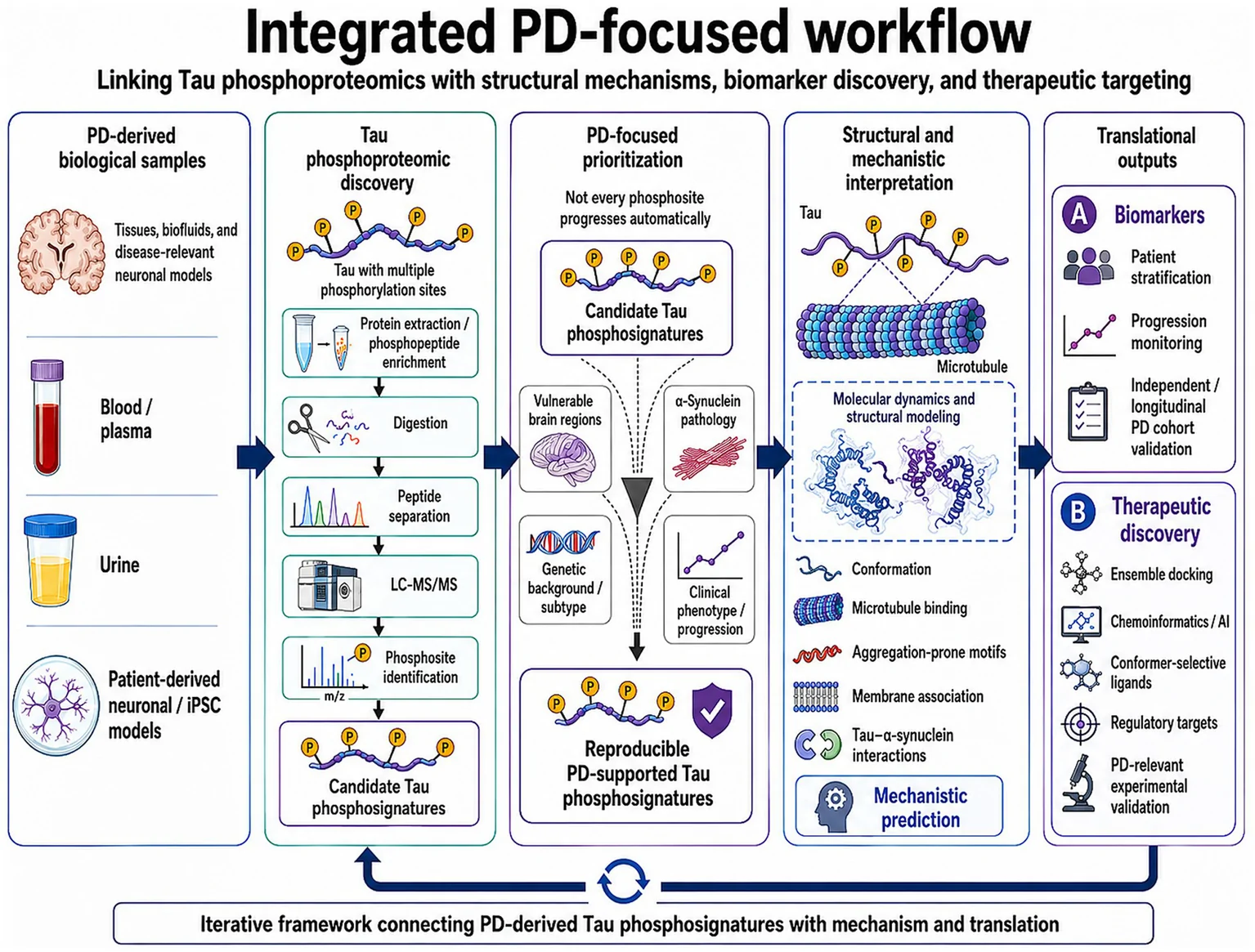
Integrated PD-focused workflow linking Tau phosphoproteomics with structural mechanisms, biomarker discovery, and therapeutic targeting. This figure provides an overview of the phosphoproteomics pipeline used to identify biomarkers and therapeutic targets in Parkinson’s disease. Biological samples—including post-mortem brain tissue, plasma or blood, urine, and cellular or iPSC-derived neuronal models—undergo protein extraction and phosphopeptide enrichment, followed by enzymatic digestion, peptide separation, and LC–MS/MS analysis. The resulting datasets are processed through bioinformatic pipelines and molecular dynamics modeling to define phosphorylation signatures, uncover therapeutic targets, inform mechanistic strategies, and guide rational drug design.

## 2 Structural organization of Tau and its interaction with tubulin

Tau is an intrinsically disordered microtubule-associated protein organized into four principal functional regions: the N-terminal projection domain, the proline-rich region, the microtubule-binding repeat region, and the C-terminal region (Mukrasch et al., 2009; Morris et al., 2011; Wang and Mandelkow, 2016). The N-terminal domain contributes to microtubule spacing, axonal organization, membrane association, and protein interactions. The adjacent proline-rich region contains numerous phosphorylation sites and signaling-protein binding motifs and also contributes to tubulin binding (Chen et al., 1992; Brandt et al., 1995; Gustke et al., 1994; Goode et al., 1997). The C-terminal region further modulates Tau conformation and intermolecular interactions (Mukrasch et al., 2007; Mukrasch et al., 2009). These functional regions are summarized in Figures 2A,B.

**Figure 2:**
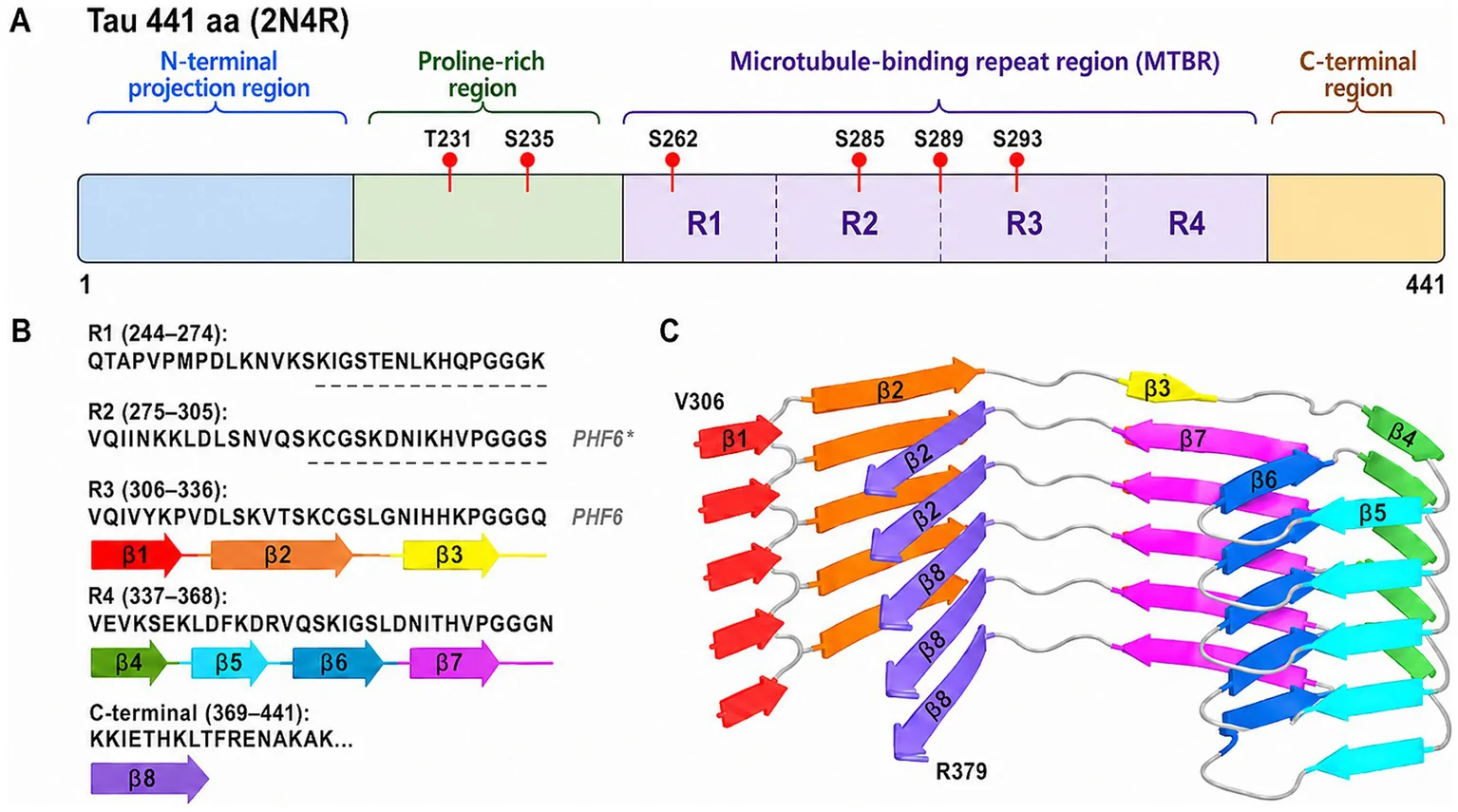
Structural foundations of phosphorylation-induced remodeling of Tau. **(A)** Functional architecture of Tau, showing the N-terminal projection region, the proline-rich region, the microtubule-binding repeat region containing R1–R4, and the C-terminal region. Representative phosphorylation sites discussed in this review are indicated: Thr231 and Ser235 in the proline-rich region, and Ser262, Ser285, Ser289, and Ser293 in the microtubule-binding repeat region. **(B)** Sequence organization and β-structure propensity of the microtubule-binding region. The sequences of repeats R1–R4 and the adjacent C-terminal segment are shown, with colored bars indicating β-strand-forming regions and gray dashed lines denoting residues capable of forming less ordered β-structure, based on sequence and structural information reported by Fitzpatrick et al. (2017). **(C)** Schematic representation of a Tau paired-helical-filament protofilament based on PDB 7UPF, showing the ordered core formed by eight β-strands (β1–β8) connected by short loops.

The microtubule-binding region constitutes the principal structural interface with tubulin and contains either three or four imperfect repeats, depending on alternative splicing of MAPT exon 10 (Goedert et al., 1989a; Goedert et al., 1989b; Lee et al., 1989; Kellogg et al., 2018). Three-repeat Tau contains R1, R3, and R4, whereas four-repeat Tau additionally contains R2. The repeats and their flanking sequences form a flexible, multivalent interface with successive α/β-tubulin heterodimers rather than a single rigid binding element (Al-Bassam et al., 2002; Kellogg et al., 2018; Brotzakis et al., 2021; Naveh-Tassa and Levy, 2026). R2 and R3 contain the amyloidogenic PHF6* (^275VQIINK^280) and PHF6 (^306VQIVYK^311) motifs, respectively (von Bergen et al., 2000; von Bergen et al., 2001; Li and Lee, 2006). Detachment from microtubules, PTMs, or conformational rearrangements can increase motif accessibility and favor oligomerization and fibril formation (Chen et al., 2019; Despres et al., 2017). Isolated repeat fragments such as R3 are therefore useful structural models but do not reproduce the long-range interactions and cooperative behavior of full-length Tau (Mukrasch et al., 2009; Melo et al., 2016).

Microtubules are assembled from α/β-tubulin heterodimers arranged longitudinally into protofilaments and laterally into a cylindrical lattice (Nogales et al., 1998; Nogales et al., 1999). Each tubulin subunit contains an N-terminal nucleotide-binding domain, an intermediate domain, and a C-terminal domain followed by a flexible acidic C-terminal tail (Nogales et al., 1998). Tau does not recognize a single, well-defined MAP-binding pocket. Its proline-rich region and R1–R4 repeats instead engage an extended microtubule-exposed surface near longitudinal interfaces between adjacent tubulin heterodimers (Butner and Kirschner, 1991; Goode et al., 1997; Kellogg et al., 2018). The acidic C-terminal tails of α- and β-tubulin provide additional contributions to this interface (Chau et al., 1998; Devred et al., 2004). Tau repeats adopt extended conformations along the protofilament and stabilize longitudinal tubulin–tubulin contacts, while adjacent Tau regions modulate complex affinity and dynamics (Fauquant et al., 2011; Lefèvre et al., 2011; Kellogg et al., 2018).

Post-translational modifications modulate this distributed interface by altering local charge and conformational accessibility. Phosphorylation, acetylation, and O-GlcNAcylation can differentially affect Tau–microtubule association in a site- and context-dependent manner (Biernat et al., 1993; Singh et al., 1996; Man et al., 2023; Liu et al., 2007; Cohen et al., 2011; Castro et al., 2023; Brister et al., 2014; Rani et al., 2020). These effects are discussed in detail in Sections 3.1 and 3.4.

### 2.1 Available experimental structures of Tau and implications for molecular docking

Because Tau is intrinsically disordered, no experimentally determined high-resolution atomic structure represents the complete conformational ensemble of soluble, full-length Tau (Mukrasch et al., 2009; Popov et al., 2019). Available structures instead capture specific states, including short or microtubule-bound fragments and ordered fibrillar cores (Kellogg et al., 2018; Fitzpatrick et al., 2017). This distinction is important: soluble Tau lacks a persistent tertiary structure, whereas pathological Tau filaments can form well-defined cross-β architectures.

Representative examples illustrate this structural diversity (Table 1). PDB 2MZ7 is an NMR ensemble of residues 267–312 in a microtubule-bound conformation and demonstrates local folding of an otherwise flexible Tau segment (Kadavath et al., 2015). Cryo-EM structures 5O3L and 5O3T resolve the ordered cores of Alzheimer’s disease paired helical and straight filaments. Structures 7UPE, 7UPF, and 7UPG capture brain-derived paired helical filaments before or during epigallocatechin-3-gallate (EGCG)-induced disassembly (Fitzpatrick et al., 2017; Seidler et al., 2022). A representative brain-derived paired-helical-filament protofilament structure (PDB 7UPF) is shown in (Figure 2C). These structures reveal ligand-accessible polar clefts within an ordered fibrillar state, but such sites should not be assumed to exist in soluble or differently modified Tau. Recombinant fibrillar structures such as 8G54/8G55 further illustrate conformational plasticity but are likewise state- and model-specific.

**Table 1:** Representative experimentally determined Tau structures available in the Protein Data Bank and their limitations for molecular docking.

PDB entry	Structural state	Tau region represented	Main relevance	Principal limitation	References
2MZ7	Microtubule-bound Tau conformation; solution NMR	Residues 267–312	Local folding of Tau upon microtubule binding	Short 46-residue fragment; the microtubule is not included in the deposited coordinates and the model does not represent full-length soluble Tau	Kadavath et al. (2015)
5O3L	Alzheimer’s disease paired helical filament; cryo-EM	Ordered core, residues 306–378	Atomic organization of the pathological PHF core	Disease- and fibril-specific conformation that does not represent soluble Tau	Fitzpatrick et al. (2017)
5O3T	Alzheimer’s disease straight filament; cryo-EM	Ordered core, residues 306–378	Alternative inter-protofilament packing of the AD Tau core	Does not represent monomeric or full-length Tau	Fitzpatrick et al. (2017)
7UPE	Brain-derived AD paired helical filament not incubated with EGCG; cryo-EM	Ordered fibrillar core	Reference fibrillar state for analysis of EGCG-induced disassembly	Only the ordered filament core is structurally resolved; no ligand-binding state is represented	Seidler et al. (2022)
7UPF/7UPG	Brain-derived AD paired helical filaments incubated with EGCG for 1 or 3 h; cryo-EM	Ordered fibrillar core with EGCG-associated polar clefts	Identification of a ligand-accessible fibrillar pharmacophore and structural intermediates of disassembly	The clefts are specific to an ordered AD filament state and should not be extrapolated to soluble Tau	Seidler et al. (2022)
8G54/8G55	Heparin-induced recombinant P2R Tau fibrils at 24 °C and 12 °C; solid-state NMR	P2R construct comprising residues 198–399; R2 and R3 form most of the ordered core	Demonstrates temperature-dependent conformational plasticity of R2 within the fibril core	Recombinant, heparin-induced and temperature-dependent fibrils; not patient-derived or representative of native soluble Tau	Mammeri et al. (2023)

Structural heterogeneity is a major limitation for conventional virtual screening and molecular docking. Tau binding cavities may be transient, shallow, and dependent on isoform, PTM pattern, aggregation state, and molecular environment (Amaro et al., 2018; Thomasen and Lindorff-Larsen, 2022; Dhar et al., 2025). Docking against a single rigid structure can therefore produce strongly conformer-dependent poses and rankings. Conformational ensembles generated by molecular dynamics or experimentally restrained modeling can partially address this limitation through ensemble docking and, where appropriate, free-energy refinement. Even then, predictions remain hypotheses for binding to a defined Tau conformer and require biochemical or biophysical validation. Representative experimental structures and their principal limitations are summarized in Table 1.

## 3 Tau as a phosphoprotein: kinases, phosphatases, and combined modifications

Reversible phosphorylation regulates Tau–microtubule association and axonal transport under physiological conditions, whereas disruption of the kinase–phosphatase balance can shift Tau toward hyperphosphorylated, structurally unstable, and aggregation-prone states (Cuchillo-Ibanez et al., 2008; Rodríguez-Martín et al., 2013; Liu et al., 2005; Liu et al., 2007). In PD and PD-relevant models, this imbalance intersects with α-synuclein pathology and genetic risk pathways, including GSK-3β, CDK5, LRRK2, and PP2A signaling (Duka et al., 2006; Wills et al., 2010; Kawakami et al., 2012; Park et al., 2016) (Figure 3).

**Figure 3:**
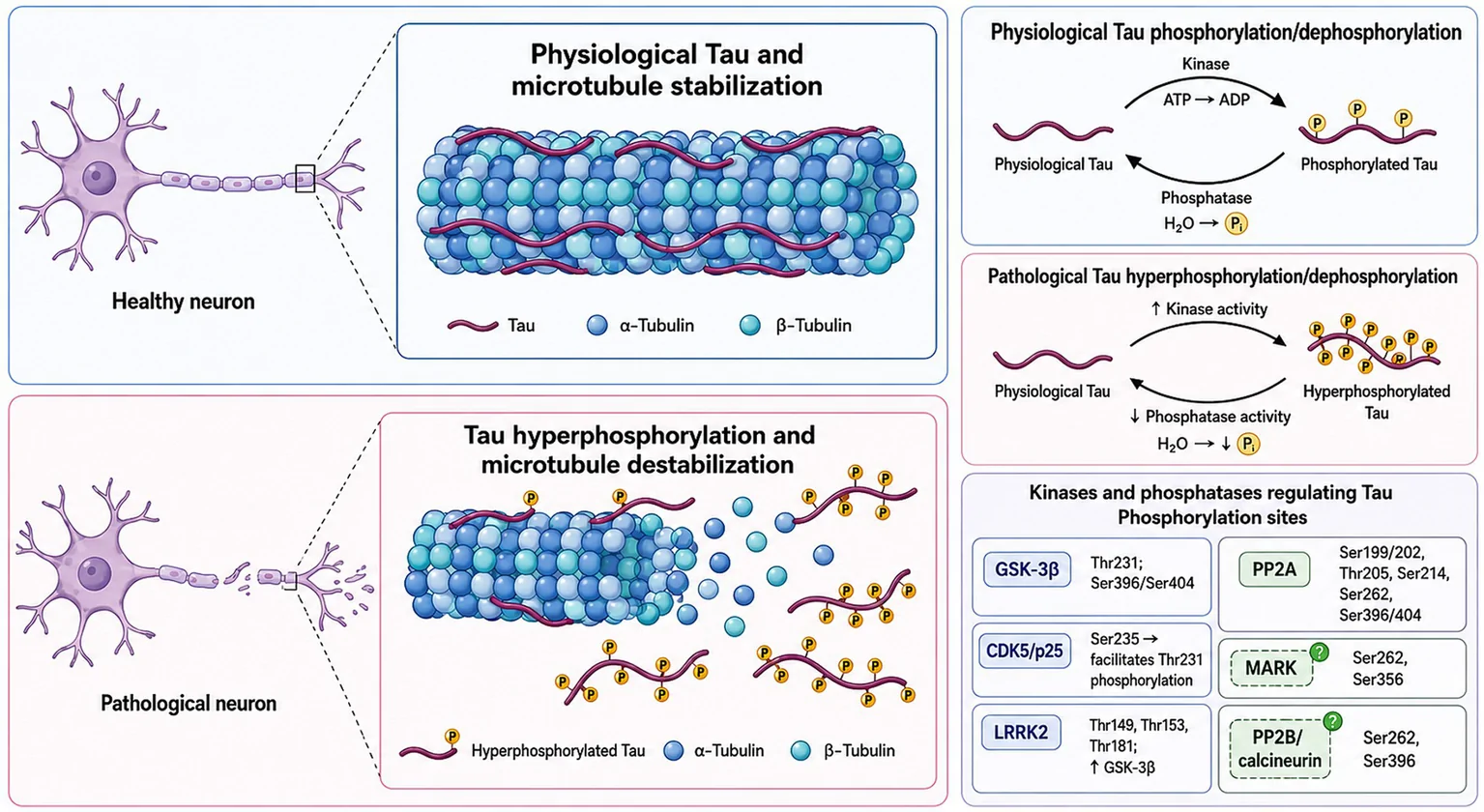
Physiological and pathological phosphorylation of Tau in microtubule regulation. This schematic compares Tau phosphorylation dynamics under physiological (top) and pathological (bottom) conditions. In healthy neurons, Tau stabilizes microtubules through a finely regulated balance between kinase and phosphatase activity. In contrast, pathological states are marked by Tau hyperphosphorylation, loss of microtubule affinity, and consequent cytoskeletal destabilization. Key kinases involved in these processes—GSK-3β, CDK5/p25, LRRK2 and MARK—are shown alongside the major phosphatases PP2A and PP2B (calcineurin). Relevant phosphorylation sites are indicated. Upward and downward arrows denote increased or reduced activity of these enzymes, respectively. Enzymes accompanied by a question mark highlight components whose contribution to Tau dysregulation in PD remains uncertain. The phosphorylation sites shown are representative and should not be interpreted as having uniform functional effects on Tau–microtubule interactions or Tau function.

### 3.1 Site-specific effects of Tau phosphorylation on microtubule binding

Tau phosphorylation does not uniformly impair microtubule interactions. Its functional consequences depend on residue position, phosphorylation stoichiometry, combinations of modified sites, and Tau structural state. Changes in direct Tau–microtubule affinity should also be distinguished from effects on Tau-mediated tubulin polymerization or microtubule stabilization (Biernat et al., 1993; Sengupta et al., 1998; Liu et al., 2007; Rodríguez-Martín et al., 2013).

Phosphorylation within the microtubule-binding region generally has inhibitory effects. Ser262 within the R1 KXGS motif markedly disrupts Tau–microtubule binding (Biernat et al., 1993). Ser356 in R4 can also reduce microtubule association, although its individual contribution is less clearly defined because Ser262 and Ser356 are frequently examined together (Singh et al., 1996; Litersky et al., 1996). Molecular dynamics simulations of an isolated R2–microtubule complex predict additional destabilization after phosphorylation at Ser289 and Ser293, with the doubly phosphorylated variant showing the lowest stability (Man et al., 2023). Thr231 phosphorylation within the proline-rich region also reduces Tau binding and microtubule-stabilizing activity, particularly when Ser235 primes subsequent GSK-3β-mediated Thr231 phosphorylation (Cho and Johnson, 2004; Sengupta et al., 2006).

Phosphorylation outside the repeat region can produce different outcomes. Under controlled *in vitro* conditions, GSK-3β-mediated phosphorylation predominantly at Ser396 and Ser404 increased Tau-mediated microtubule assembly and association; this effect diminished as additional proline-rich residues became phosphorylated (Liu et al., 2007). Ser396/Ser404 phosphorylation should therefore not be interpreted as universally affinity-enhancing.

Overall, the evidence supports a site- and context-dependent model. Restricted phosphorylation can preserve or enhance selected Tau functions, whereas phosphorylation of key repeat or proline-rich residues and broader multisite hyperphosphorylation favor Tau detachment and cytoskeletal destabilization (Biernat et al., 1993; Sengupta et al., 1998; Liu et al., 2007).

### 3.2 Key kinases in Tau phosphorylation

Several kinases connect Tau phosphorylation with PD-relevant molecular pathways (Table 2). GSK-3β is among the best characterized. In neurotoxin-based PD models using 1-methyl-4-phenylpyridinium (MPP+) in cellular systems or its precursor 1-methyl-4-phenyl-1,2,3,6-tetrahydropyridine (MPTP) *in vivo*, α-synuclein enhances GSK-3β activation and GSK-3β-dependent Tau phosphorylation (Duka et al., 2006; Duka et al., 2009; Kawakami et al., 2011). The site-specific functional consequences of GSK-3β-mediated Tau phosphorylation are detailed in Section 3.1 (Cho and Johnson, 2004; Liu et al., 2007).

**Table 2:** Principal kinases and phosphatases implicated in Tau phosphorylation in Parkinson’s disease and PD-relevant experimental models.

Protein	Type	Primary Tau residues targeted	Relevance to Parkinson’s disease	References
GSK-3β	Kinase	Ser202, Thr231, Ser396, Ser404	One of the most extensively studied Tau kinases in PD. Increased active GSK-3β and regional Tau phosphorylation have been reported in post-mortem PD striatum and PD-relevant models. Its functional effects are site- and context-dependent: Thr231 phosphorylation reduces Tau–microtubule binding, whereas restricted C-terminal phosphorylation predominantly at Ser396/Ser404 may preserve or modestly enhance Tau-mediated microtubule assembly under defined *in vitro* conditions. Broader multisite phosphorylation is associated with Tau dysfunction and cytoskeletal destabilization.	Cho and Johnson (2004); Liu et al. (2007); Wills et al. (2010)
CDK5/p25	Kinase	Ser202/Thr205, Ser235, Ser404	Cellular stress can induce calpain-mediated cleavage of the CDK5 activator p35 into p25, resulting in prolonged and mislocalized CDK5 activity. CDK5/p25 phosphorylates Tau at several sites and promotes cytoskeletal disruption and neuronal injury. CDK5 activity contributes to dopaminergic neuron loss in MPTP models, although Tau phosphorylation has not been established as the sole mediator of this effect.	Patrick et al. (1999); Lee et al. (2000); Hashiguchi et al. (2002); Smith et al. (2003)
LRRK2	Kinase	Thr149, Thr153, Thr181	Pathogenic LRRK2 variants are among the most common genetic causes of PD. LRRK2 can directly phosphorylate Tau, particularly tubulin-associated Tau, and can increase GSK-3β activity, thereby linking genetic susceptibility to Tau phosphoregulatory dysfunction.	Bailey et al. (2013); Kawakami et al. (2012); Kawakami et al. (2014)
MARK	Kinase	Ser262, Ser356	MARK family kinases phosphorylate KXGS motifs within the microtubule-binding region, reducing Tau–microtubule affinity and promoting microtubule disruption. Their direct contribution to Tau pathology in PD remains incompletely defined, and most mechanistic evidence derives from general tauopathy models.	Drewes et al. (1997); Sultanakhmetov et al. (2024)
PP2A	Phosphatase	Ser199/202, Thr205, Ser214, Ser262, Ser396/404	PP2A accounts for approximately 71% of the Tau phosphatase activity measured in human brain extracts and acts on multiple pathological epitopes. PD and DLB brains show dysregulation of PP2A methylation and a lower proportion of its active methylated form, but total PP2A expression was not reduced and the direct consequences for Tau phosphorylation in PD remain unresolved.	Sontag et al. (1996); Liu et al. (2005); Park et al. (2016)
PP2B (calcineurin)	Phosphatase	Ser262, Ser396	A calcium/calmodulin-regulated phosphatase linking calcium signaling to Tau dephosphorylation. Human-brain preparations show preferential dephosphorylation of Tau at Ser262 and Ser396. Its specific contribution to Tau dysregulation in PD remains uncertain.	Liu et al. (2005); Rahman et al. (2006)

CDK5 provides another link between cellular stress and Tau dysregulation. Calpain-dependent conversion of p35 to p25 produces prolonged CDK5 activity, which can promote Tau hyperphosphorylation and neuronal injury (Patrick et al., 1999; Lee et al., 2000). Increased CDK5 activity contributes to dopaminergic neuron loss in MPTP models, although Tau phosphorylation has not been established as the sole mediator (Smith et al., 2003). CDK5 can also prime GSK-3α/β activity through Ser235-dependent facilitation of Thr231 phosphorylation (Sengupta et al., 2006).

Genetic PD further implicates LRRK2, which can phosphorylate Tau at Thr149, Thr153, and Thr181 and enhance GSK-3β activity (Bailey et al., 2013; Kawakami et al., 2012; Kawakami et al., 2014). MARK-family kinases target KXGS motifs, including Ser262 and Ser356, thereby promoting Tau dissociation from microtubules, although their direct contribution to Tau pathology in PD remains incompletely defined (Drewes et al., 1997; Sultanakhmetov et al., 2024). Other pathways, including PKC signaling, may indirectly modulate Tau regulation through GSK-3β, but their direct relevance to Tau phosphorylation in PD is less established (Isagawa et al., 2000; Gordon et al., 2016).

Together, these findings indicate an overlapping kinase network rather than a single dominant pathway, which complicates therapeutic inhibition because physiological kinase functions and pathway redundancy must be preserved.

### 3.3 Phosphatases counteracting Tau hyperphosphorylation

PP2A is the principal Tau phosphatase in human brain extracts, accounting for approximately 71% of measured Tau phosphatase activity and acting on multiple epitopes, including Ser199/202, Thr205, Ser214, Ser262, and Ser396/404 (Liu et al., 2005; Sontag et al., 1996). PD and DLB brains show altered PP2A methylation and a lower proportion of its active methylated form. These findings have been linked primarily to α-synuclein regulation, and their direct contribution to Tau hyperphosphorylation in PD remains unresolved (Park et al., 2016).

PP2A activity can also be modified by endogenous inhibitors and phosphorylation-dependent mechanisms. Src-dependent regulation has been implicated in Tau phosphorylation, although recent work challenges Tyr307 as a universal inhibitory site and identifies Tyr127 and Tyr284 as alternative Src/Fyn-regulated residues (Xiong et al., 2013; Sontag et al., 2022). Experimental PP1/PP2A inhibition promotes Tau hyperphosphorylation and pathological conformational changes. Conversely, enhancing PP2A-related activity with sodium selenate reduces Tau pathology in transgenic tauopathy models, providing mechanistic evidence rather than direct demonstration of therapeutic benefit in PD (Zhang and Simpkins, 2010; van Eersel et al., 2010).

PP2B/calcineurin, PP1, and PP5 provide additional Tau phosphatase activity. Human-brain preparations show preferential PP2B-mediated dephosphorylation at Ser262 and Ser396, whereas PP1 and PP5 contribute substantially less total Tau phosphatase activity than PP2A (Rahman et al., 2006; Liu et al., 2005). Overall, altered kinase–phosphatase balance remains a plausible component of Tau dysregulation in PD, but evidence that restoring Tau-directed phosphatase activity modifies PD progression is currently limited.

### 3.4 Crosstalk among Tau post-translational modifications: charge-dependent effects on structure and aggregation

Tau phosphorylation functions within a broader network of post-translational modifications. Phosphorylation introduces negatively charged phosphate groups that alter local electrostatic interactions and can promote new phosphate–lysine or phosphate–arginine contacts; however, the resulting structural effects remain strongly dependent on phosphosite identity, stoichiometry, and molecular context. O-GlcNAcylation, added by O-GlcNAc transferase and removed by O-GlcNAcase, can compete with phosphorylation at the same or nearby serine and threonine residues (Hart et al., 2011; Van der Laarse et al., 2018; Bourré et al., 2018). In Alzheimer’s disease brain, reduced O-GlcNAcylation has been associated with impaired glucose metabolism and increased Tau phosphorylation (Liu et al., 2009). Reduced global O-GlcNAcylation has also been reported in post-mortem PD substantia nigra, although its direct contribution to Tau hyperphosphorylation remains unknown (Kim et al., 2026). Experimental tauopathy models support a protective mechanism: O-GlcNAcylation at Ser400 reduces PHF-1 phosphorylation and fibrillar assembly, whereas pharmacological OGA inhibition reduces Tau aggregation and neurodegeneration (Yuzwa et al., 2012; Yuzwa et al., 2014; Cantrelle et al., 2021).

Acetylation acts differently by neutralizing positively charged lysine residues. Acetylation at K274, K280, and K281 can weaken Tau–microtubule interactions. Its aggregation effects are also site-dependent: K280 acetylation promotes β-sheet formation and dimerization, whereas K321 acetylation can reduce β-sheet organization and Tau self-association (Cohen et al., 2011; Castro et al., 2023; Zou et al., 2024).

These findings support a combinatorial PTM model in which phosphorylation, acetylation, and O-GlcNAcylation may reinforce or oppose one another. Their structural consequences depend on residue position, stoichiometry, and molecular context rather than net charge alone; the corresponding molecular-dynamics mechanisms are discussed in Section 6.

### 3.5 Phosphorylation cross-talk between Tau and α-synuclein

Tau and α-synuclein are connected through overlapping phosphorylation networks. α-Synuclein Ser129 is the predominant phospho-epitope in Lewy pathology and is regulated by several kinases and PP2A (Fujiwara et al., 2002; Ishii et al., 2007; Mbefo et al., 2010; Hara et al., 2013; Park et al., 2016). Its functional significance remains context-dependent. Some PD models associate Ser129 phosphorylation with increased toxicity, whereas other evidence indicates that it may occur after deposition and reduce seeded fibril formation (Chen and Feany, 2005; Ghanem et al., 2022). Thus, α-synuclein pSer129 is a robust pathological marker but should not be regarded as uniformly toxicity-promoting.

α-Synuclein can bind Tau and stimulate PKA-dependent phosphorylation at Ser262 and Ser356 (Jensen et al., 1999). MPP+/MPTP and other PD-relevant models also show α-synuclein-facilitated GSK-3β-dependent Tau phosphorylation, including Ser396/404 (Duka et al., 2006; Duka et al., 2009; Kawakami et al., 2011). In the A53T α-synuclein mouse model, increased Tau phosphorylation at Ser202, Ser262, and Ser396/404 was accompanied by increased active GSK-3β, paralleling alterations in post-mortem PD striatum (Wills et al., 2010; Wills et al., 2011). These changes may weaken Tau–microtubule interactions, while direct Tau–α-synuclein association can separately promote co-aggregation and cross-seeding (Giasson et al., 2003; Badiola et al., 2011; Padilla-Godínez et al., 2025). PP2A is an additional shared regulatory node, although coordinated phosphatase regulation of both proteins within the same PD system remains incompletely characterized (Liu et al., 2005; Park et al., 2016).

A major unresolved question is whether coordinated Tau and α-synuclein phosphoregulation contributes directly to clinical progression in PD. Joint phosphosite profiling in PD tissues, biofluids, and patient-derived neuronal models may help identify reproducible molecular subgroups. Structural and computational predictions should then be validated in PD-relevant systems because much of the available mechanistic evidence derives from Alzheimer’s disease or general tauopathy models.

## 4 Studying the phosphoproteome in PD tissues

In PD, phosphoproteomics should move beyond cataloging phosphorylation sites toward identifying reproducible Tau phosphosignatures. Priority signatures are those associated with vulnerable nigrostriatal regions, dopaminergic neurons, α-synuclein pathology, genetic subtype, clinical phenotype, or disease progression (Wills et al., 2010; Bogetofte et al., 2023; Rutledge et al., 2024). Such experimentally supported signatures provide the biological starting point for subsequent structural and therapeutic analyses.

### 4.1 Post-mortem analysis and spatially resolved proteomic methods

Post-mortem human brain provides direct access to the regional and cellular environments affected in PD, but bulk proteomics averages signals across heterogeneous anatomical regions and cell populations. This limitation is particularly relevant because molecular alterations vary among brain regions and dopaminergic neuronal subtypes (Kamath et al., 2022; Jang et al., 2023; Zhu et al., 2024). Spatially resolved approaches, including laser-capture microdissection coupled to mass spectrometry and imaging mass spectrometry, can preserve anatomical information while enabling molecular profiling (Aichler and Walch, 2015; Griesser et al., 2020). LCM-based workflows have quantified more than 5,600 protein groups from microdissected human substantia nigra, demonstrating feasibility in small, anatomically defined samples, although phosphosite-resolved spatial datasets from human PD brain remain limited (Griesser et al., 2020).

Probe-dependent proximity profiling provides a complementary strategy. Using the AT8 antibody against pSer202/pThr205 Tau, ProPPr identified proteins surrounding phospho-Tau aggregates in Alzheimer’s disease, corticobasal degeneration, Pick’s disease, and progressive supranuclear palsy (Morderer et al., 2025). Because PD was not included and proximity labeling does not establish direct physical interaction, these findings should be regarded as a methodological precedent rather than evidence of Tau-associated networks in PD. Adaptation of this approach using PD-relevant Tau or α-synuclein phospho-epitopes could help define lesion-associated molecular environments.

Deep phosphoproteomic workflows combining phosphopeptide enrichment with quantitative high-resolution LC–MS/MS can detect tens of thousands of phosphorylation sites. Alzheimer’s disease studies have demonstrated this analytical depth, including detection of more than 34,000 phosphosites, but such datasets remain methodological precedents rather than evidence of PD-specific phosphoregulation (Bai et al., 2020). Application to PD will require adequately powered cohorts, control of post-mortem variables, and replication across brain regions and analytical platforms.

### 4.2 Phosphoproteome in liquid biopsies: blood, plasma, cerebrospinal fluid, and urine

Biofluids complement post-mortem tissue by enabling repeated sampling across disease stages. Plasma is minimally invasive, whereas cerebrospinal fluid more directly reflects the central nervous system but requires invasive collection (Rutledge et al., 2024). Large-scale proteomic studies demonstrate the feasibility of longitudinal and consortium-based biomarker discovery in PD. For example, Hällqvist et al. (2024) identified an eight-protein plasma panel associated with established and prodromal PD, although it did not contain Tau phosphopeptides. Similarly, large collaborative initiatives involving plasma, serum, and CSF illustrate the value of harmonized datasets for replication and disease stratification (Imam et al., 2025).

Urinary extracellular vesicles provide an additional non-invasive source and have yielded PD-associated proteins and phosphoproteins related to autophagy, neuroinflammation, and neuronal injury (Hadisurya et al., 2023). However, evidence that urine contains informative brain-derived Tau phosphosignatures remains limited. More generally, most current liquid-biopsy studies measure protein abundance rather than site-specific Tau phosphorylation. Mass-spectrometric studies in Alzheimer’s disease further demonstrate that Tau phosphorylation is compartment- and site-specific, with differences between brain and CSF and with only selected CSF Tau sites showing hyperphosphorylation (Barthélemy et al., 2019; Barthélemy et al., 2021). Phosphopeptide enrichment coupled to mass spectrometry may increase sensitivity to kinase–phosphatase dysregulation, but validated circulating Tau phosphosignatures for PD have not yet been established.

Cross-validation across brain tissue, biofluids, and disease-relevant neuronal models will therefore be essential for distinguishing reproducible PD-associated phosphosignatures from compartment-specific or nonspecific changes. Candidate Tau phosphosites and related molecular features are summarized in Table 3.

**Table 3:** Key Tau-related phosphosites and interacting proteins in Parkinson’s disease: functional and therapeutic insights.

Phosphoprotein or molecular feature	Biological function	Detection method and evidence in PD	Implication for PD pathogenesis	Relevance for structural modeling	References
Tau pSer262 and pSer396/404	Ser262 phosphorylation directly weakens Tau–microtubule binding. The effects of Ser396/404 phosphorylation are context-dependent, although these epitopes are frequently associated with pathological multisite phosphorylation.	Increased pSer262 and pSer396/404 were detected by biochemical analyses of post-mortem PD and PDD striatum. Similar alterations have been reproduced in α-synuclein-dependent MPTP models.	Supports a regionally restricted α-synuclein–GSK-3β–Tau pathway in PD. However, these sites alone should not be regarded as sufficient drivers of Tau pathology.	Defined phosphoconformers can be modeled to examine microtubule binding, Tau self-association, and Tau–α-synuclein interactions. MD cannot independently establish PD specificity.	Biernat et al. (1993); Duka et al. (2006); Liu et al. (2007); Wills et al. (2010)
α-Synuclein pSer129	Predominant phospho-epitope in Lewy pathology. It influences α-synuclein localization, turnover, membrane association, inclusion dynamics, and seeding in a context-dependent manner.	Detected in human Lewy body disease tissue using biochemical, immunological, and mass-spectrometric approaches, and studied extensively in cellular and seeded experimental models.	Serves as a robust marker of Lewy pathology. Its direct effects on aggregation and toxicity remain dependent on the experimental model and stage of deposition.	Defined pSer129 states can be examined for effects on membrane association, oligomerization, neutralization fibril surfaces, and Tau–α-synuclein interfaces.	Anderson et al. (2006); Ghanem et al. (2022); Padilla-Godínez et al. (2025)
LRRK2 pathway: LRRK2 pSer935 and Tau pThr149/pThr153/pThr181	LRRK2 pSer935 participates in 14–3-3 binding and cellular localization but is not a direct measure of intrinsic kinase activity. LRRK2 can directly or indirectly increase Tau phosphorylation at Thr149, Thr153, Thr181, and additional sites.	LRRK2 pSer935 has been measured in post-mortem PD brain and peripheral blood cells as a pathway and target-engagement marker. Tau phosphorylation sites were identified mainly through biochemical, cellular, and transgenic-model studies.	Pathogenic LRRK2 activity may contribute to Tau phosphoregulatory dysfunction. The functional meaning of pSer935 should be distinguished from the direct phosphorylation of Tau or Rab substrates.	Supports modeling of LRRK2–14–3-3 interactions, inhibitor-dependent LRRK2 conformations, and the separate structural consequences of LRRK2-associated Tau phosphosites.	Nichols et al. (2010); Kawakami et al. (2012); Bailey et al. (2013); Dzamko et al. (2017); Vissers et al. (2023)
AT8-positive pTau proximity proteome	Includes proteins located near phospho-Tau lesions, with functions related to trafficking, lysosomal biology, kinase signaling, iron handling, stress responses, and proteostasis. Reported candidates include VPS35, LAMP2, GSK3α, FTL, and VGF.	Identified by ProPPr in post-mortem human tissue from AD, CBD, PiD, and PSP. Direct PD evidence is not yet available.	Provides transferable candidates for investigating Tau-associated molecular environments in PD. Proximity does not demonstrate direct binding or a causal role.	Can prioritize candidate Tau–protein interfaces for subsequent biochemical and structural validation, but ProPPr findings alone should not be used as direct docking templates.	Morderer et al. (2025)

## 5 Phosphoproteome in cellular models and neuronal cultures

Cellular models are essential for understanding how Tau phosphorylation is regulated and dysregulated in PD. They provide controlled environments in which specific molecular pathways can be manipulated and offer mechanistic insights that cannot be obtained from post-mortem tissue alone. Neuronal cell lines contribute valuable preliminary data, but dopaminergic neurons derived from patient-specific induced pluripotent stem cells (iPSCs) have become particularly important. These human-derived models preserve genetic background, capture disease-relevant vulnerabilities, and serve as a critical bridge between basic discovery and translational research (Bose et al., 2022).

### 5.1 Experimental strategies and mechanistic insights

PD-relevant cellular and animal models link mitochondrial stress and α-synuclein to Tau phosphorylation. In primary mesencephalic neuronal cultures and MPTP-treated mice, MPP + and MPTP, respectively, increased Tau Ser396/Ser404 phosphorylation in an α-synuclein-dependent manner; subsequent studies implicated GSK-3β at Ser262 and Ser396/Ser404 (Duka et al., 2006; Duka et al., 2009). Bidirectional Tau–α-synuclein effects have also been observed in cellular systems (Grassi et al., 2019; Badiola et al., 2011; Padilla-Godínez et al., 2025). These targeted studies provide mechanistic rather than comprehensive phosphoproteomic evidence and do not establish a self-sustaining cycle that determines clinical PD progression.

### 5.2 iPSC models and genetic subtypes

Patient-derived iPSC models provide an important human genetic context for investigating Tau dysregulation, although the observed phenotypes should not be generalized across PD-associated variants. In iPSC-derived neurons from patients carrying the I2020T LRRK2 mutation, reduced AKT signaling and increased Tau phosphorylation through the AKT/GSK-3β pathway were reported (Ohta et al., 2015). This finding is specific to the mutation and experimental system examined and does not establish increased Tau phosphorylation as a shared feature of all LRRK2-associated PD.

Direct phosphoproteomic evidence has been obtained from patient-derived dopamine neurons carrying the GBA-N370S variant. A multi-part enrichment proteomic and post-translational-modification workflow identified extensive changes in proteins and PTMs, impaired neurite outgrowth and mitochondrial movement, and Tau as a functional mediator within the affected molecular network (Bogetofte et al., 2023). This study demonstrates the value of PTM-resolved analyses in patient-derived PD models, but it does not establish a universal GBA-associated Tau phosphosignature or a relationship between the identified modifications and clinical severity or progression.

Cross-validation across patient-derived neurons, vulnerable brain regions, and accessible biofluids will be necessary to distinguish reproducible PD-associated phosphosignatures from genotype-, cell-type-, or model-specific effects. Replicated Tau phosphosites can then be prioritized for structural analysis, phosphosite-specific biomarker assays, and experimental evaluation of the corresponding regulatory pathways.

## 6 Molecular dynamics of Tau post-translational modifications: structural mechanisms and functional consequences

Molecular dynamics (MD) simulations are particularly suited to Tau because its intrinsically disordered conformational ensemble is highly sensitive to post-translational modifications. MD can resolve residue-level changes in electrostatics, hydrogen bonding, secondary-structure propensity, compactness, self-association, microtubule binding, and membrane interactions under defined molecular conditions (Mukrasch et al., 2009; Brotzakis et al., 2021).

These simulations provide mechanistic hypotheses rather than evidence that a modeled Tau state is specific to PD. Their interpretation depends on the Tau construct, modification pattern, aggregation state, starting conformation, force field, sampling strategy, and simulation resolution. Moreover, many available studies use isolated Tau fragments or structures derived from Alzheimer’s disease and other tauopathies. Representative studies and their principal structural findings are summarized in Table 4.

**Table 4:** Representative molecular dynamics studies of the structural and functional effects of Tau post-translational modifications.

Modification	Tau system and variants analyzed	Principal structural and functional findings	Reference
Phosphorylation	Dimeric models of Tau 254–290 containing the KXGS and VQIINK motifs; non-modified Tau, Δ280 positive control, pSer262, and triple-phosphorylated pSer262/pSer285/pSer289 variants	The non-modified and pSer262 dimers were conformationally unstable and intermittently dissociated. By contrast, triple phosphorylation stabilized compact dimers with more favorable interaction energies, resembling the behavior of the aggregation-prone Δ280 positive control.	Alipour et al. (2022)
Phosphorylation	Microtubule-bound Tau R2 repeat (275–305) and soluble R2 peptides; non-modified, pSer289, pSer293, and pSer289/pSer293 variants	Phosphorylation at Ser289, Ser293, or both residues weakened the predicted R2–microtubule interaction. In soluble R2 peptides, phosphorylation also increased helix-to-coil transitions, although the relationship between these local changes and full-length Tau aggregation requires experimental validation.	Man et al. (2023)
Phosphorylation	Tau 225–250 within the proline-rich region; non-modified, pThr231, pSer235, pThr231/pSer235, truncated variants, and alternative phosphate charge states	All phosphorylation patterns disrupted a nascent terminal β-sheet involving the VAVVR and QTAPVP regions. Dual pThr231/pSer235 phosphorylation under physiological ionic conditions produced a transient α-helix and showed the closest agreement with available NMR data, whereas polyproline II conformations occurred only sporadically.	Lyons et al. (2014)
Phosphorylation and repeat-specific mutations	Narrow Pick filament models: NPF-WT, NPF-PS phosphorylated at Ser262/Ser324/Ser356, NPF-GG carrying E264G/D358G, and a repeat-specific G326E variant	Phosphorylation and repeat-specific substitutions altered local electrostatic interactions and the conformation of the PGGG motifs. E264G shifted the R1 PGGG motif from a compact turn toward a less ordered conformation, while homologous sequence changes in R3 produced different effects. The results demonstrate dependence on the pre-existing Pick’s disease-specific fold and should not be extrapolated directly to PD.	Sahayaraj et al. (2023)
Phosphorylation and acetylation	Computational models of full-length 2N4R Tau; phosphorylated pTau1, pTau2, and pTau3 analogs and eight acetylated variants involving K163, K180, K240, K274, K280, K281, K294, K317, and K369; examined in solution and near a microtubule model	Increasing negative charge in phosphorylated variants and neutralization of lysine charge in acetylated variants altered Tau electrostatics and conformation. In most models, these changes reduced accessibility of the microtubule-binding region or hindered the predicted association with tubulin. The variants were computationally designed from phosphorylation patterns described in normal and Alzheimer’s disease brains.	Castro et al. (2023)
Phosphorylation versus O-GlcNAcylation	Tau 225–246 within the P2 proline-rich region; non-modified Tau, Tau2p (pThr231/pSer235), Tau4p (pThr231/pSer235/pSer237/pSer238), and O-GlcNAcylated Thr231 and Ser235 variants	Multisite phosphorylation promoted intramolecular phosphate–lysine/arginine salt bridges, collapsed conformations, and disruption of the local antiparallel β-sheet. O-GlcNAcylation produced more modest structural changes and retained extended conformations resembling the non-modified peptide.	Rani et al. (2020)
Phosphorylation	Tau 256–267 within R1; non-modified and pSer262 variants analyzed in microtubule-bound complexes and as soluble peptides	Ser262 phosphorylation reduced predicted Tau–microtubule binding by disrupting hydrogen bonds and decreasing the contact area. In the soluble peptide, it favored a slightly more expanded ensemble and reduced self-association by weakening pairwise interactions and the K259–E264 salt bridge.	Xu et al. (2025)
Acetylation	Dimers of the R1–R4 microtubule-binding repeats, with detailed analysis of non-modified and K280-acetylated R2 and non-modified and K321-acetylated R3	R3 displayed the greatest intrinsic dimerization and β-structure propensity among the non-modified repeats. K280 acetylation increased β-sheet formation and strengthened R2 interchain interactions, whereas K321 acetylation reduced R3 β-sheet content, shortened β-strands, and favored helical conformations.	Zou et al. (2024)
Acetylation	Microtubule-bound Tau R2 repeat; non-modified, K274ac, K280ac, and K281ac variants	Acetylation at all three sites weakened the predicted stability and binding affinity of the Tau R2–microtubule complex. K280ac produced the largest predicted loss of binding, while K274ac and K281ac predominantly disrupted contacts within the C-terminal portion of R2. Acetylation also promoted a more compact intramolecular R2 conformation.	Xia et al. (2026)

ac, acetylated; MT, microtubule; NPF, narrow Pick filament; O-GlcNAc, O-linked β-N-acetylglucosamine; p, phosphorylated; R1–R4, microtubule-binding repeats 1–4; WT, wild type or non-modified Tau.

### 6.1 Phosphorylation-induced structural remodeling and microtubule dissociation

Building on the site-specific functional effects summarized in Section 3.1, MD studies show that Tau phosphorylation produces context-dependent structural responses rather than a uniform transition toward aggregation. In the 225–250 proline-rich segment, Thr231/Ser235 phosphorylation reorganized local electrostatic interactions, disrupted a transient β-sheet, and altered the populations of turns, α-helical, and polyproline II-like conformations. Because this study examined an isolated peptide rather than a Tau–microtubule complex, its implications for microtubule binding remain mechanistic inferences (Lyons et al., 2014).

Within the microtubule-binding repeats, phosphorylation can directly destabilize Tau–tubulin contacts. In R2, phosphorylation at Ser289 or Ser293 reduced predicted microtubule affinity, with the double-phosphorylated form showing the greatest destabilization (Man et al., 2023). Ser262 provides a complementary example: phosphorylation disrupted hydrogen bonds and reduced the Tau–microtubule contact area, whereas simulations of the corresponding soluble peptide favored a somewhat more expanded ensemble with reduced self-association. Thus, microtubule dissociation does not necessarily imply an immediate increase in intrinsic aggregation propensity (Xu et al., 2025).

Multisite phosphorylation can generate cooperative effects that differ from those of individual residues. In Tau 254–290 dimers, phosphorylation at Ser262 alone produced relatively unstable complexes, whereas combined phosphorylation at Ser262, Ser285, and Ser289 stabilized more compact assemblies resembling the behavior of an aggregation-prone Δ280 control (Alipour et al., 2022). Similarly, computational models of full-length 2N4R Tau showed that more extensively phosphorylated variants adopted more compact conformations and displayed greater impairment of predicted tubulin association than moderately phosphorylated variants (Castro et al., 2023).

Overall, phosphorylation within the microtubule-binding region generally weakens predicted Tau–tubulin interactions, but soluble Tau can shift toward either expanded, less self-associated states or compact multimolecular assemblies. These outcomes depend on phosphosite identity, combinations of sites, and molecular context. Because several modeled phosphorylation patterns derive from Alzheimer’s disease or non-PD systems, they should not be interpreted as PD-specific phosphoconformers.

### 6.2 Molecular interplay between phosphorylation, acetylation, and O-GlcNAcylation

MD simulations illustrate how different PTMs can generate opposing effects within the same Tau region. In the 225–246 proline-rich segment, multisite phosphorylation promoted conformational collapse through intramolecular phosphate–lysine and phosphate–arginine salt bridges and disrupted a local antiparallel β-sheet. O-GlcNAcylation at Thr231 and Ser235 instead produced more modest structural changes and maintained more extended conformations through altered hydration and hydrogen-bonding interactions (Rani et al., 2020).

Acetylation acts through lysine-charge neutralization but is similarly site-dependent. Full-length 2N4R simulations predicted altered electrostatics and impaired microtubule association in variants involving K274, K280, and K281 (Castro et al., 2023). In repeat dimers, K280 acetylation increased β-sheet formation and interchain interactions in R2, whereas K321 acetylation reduced β-sheet content and promoted more helical conformations in R3 (Zou et al., 2024). These contrasting outcomes reinforce a combinatorial model in which the structural effects of PTMs depend on residue position and conformational context rather than modification type alone.

Available simulations were not based on PD-derived Tau structures or PD-specific PTM patterns; they therefore provide transferable mechanisms that require validation in PD-relevant systems.

### 6.3 Tau aggregation and interactions with neuronal membranes

Experimental studies show that Tau associates preferentially with anionic membranes, where lipid binding can promote Tau self-assembly and, conversely, Tau oligomers can perturb membrane organization (Ait-Bouziad et al., 2017; Mari et al., 2018). MD simulations provide a structural framework for these interactions and identify three major determinants: membrane composition, Tau fragment length and assembly state, and fibril architecture (Cheng et al., 2022; Nguyen and Derreumaux, 2022; Chowdhury et al., 2023).

Representative simulations illustrate these determinants. Tau-K18 preferentially associated with boundaries between ordered and disordered lipid domains and with GM1- or phosphatidylserine-enriched regions; membrane-bound oligomers also perturbed local lipid organization (Cheng et al., 2022). In an Alzheimer’s disease-derived R3–R4 model, oligomerization altered the location and depth of Tau–lipid contacts (Nguyen and Derreumaux, 2022). Alzheimer’s disease-derived paired helical filaments also formed more extensive lipid contacts and greater membrane compaction than straight filaments (Chowdhury et al., 2023). These models provide mechanistic insight but do not reproduce soluble full-length PD-specific Tau.

Simulation adequacy should be judged by convergence and reproducibility of question-relevant observables rather than by a universal trajectory length. The studies in Table 5 range from hundreds of nanoseconds in atomistic simulations to ≥15 μs in coarse-grained lipid-raft systems. Coarse-grained approaches such as Martini extend accessible spatial and temporal scales and can identify membrane-binding events, preferred lipid domains, and large-scale reorganization, but they sacrifice atomistic detail (Marrink et al., 2007; Monticelli et al., 2008). Multiscale workflows combining coarse-grained sampling with atomistic refinement, independent replicates, convergence analysis, and experimental validation are therefore particularly useful for intrinsically disordered Tau (Cheng et al., 2022; Wassenaar et al., 2014; Thomasen et al., 2022).

**Table 5:** Molecular dynamics simulations of Tau–membrane interactions.

Membrane system	Tau construct and assembly state	Simulation approach	Principal mechanistic findings	Reference
Heterogeneous lipid-raft models containing cholesterol-rich, GM1-enriched, or phosphatidylserine-enriched domains	Tau-K18 fragment; monomeric, dimeric, and tetrameric states	Martini 2.20 coarse-grained MD with 15-μs trajectories, followed by selected 200-ns all-atom refinement using AMBER99SB for Tau and the SLIPID force field for lipids	Tau species preferentially associated with boundaries between liquid-ordered and liquid-disordered domains and interacted strongly with GM1- and phosphatidylserine-enriched regions. Membrane-bound oligomers disrupted local lipid-chain order, and transient β-sheet formation was observed in selected systems.	Cheng et al. (2022)
Anionic DOPC:DOPS bilayer (1:1)	Tau R3–R4 fragment, residues 306–378; monomeric and dimeric states	All-atom MD using CHARMM36m in GROMACS with 150 mM KCl; trajectories extending to approximately 2 μs	The monomer showed deep insertion of the PHF6 motif and adjacent residues. The dimer retained a propensity to form β-hairpin-like conformations but displayed a different interaction mode, characterized by insertion of the C-terminal R4 region and transient adsorption of the PHF6 motif. Thus, Tau assembly state altered the distribution and depth of membrane contacts.	Nguyen and Derreumaux (2022)
Symmetric six-component neuronal membrane model containing POPC, cholesterol, GM1, POPS, POPE, and sphingomyelin	Alzheimer’s disease-derived paired helical filaments and straight filaments	All-atom MD using CHARMM36m for Tau and CHARMM36 for lipids in GROMACS; 1-μs membrane-associated trajectories and 500-ns aqueous control simulations	Both filament types were more compact in the membrane environment than in water. Paired helical filaments approached the membrane more closely, established more extensive fibril–lipid contacts, and produced greater lipid compaction than straight filaments. The simulations captured early fibril–membrane association rather than complete insertion or permeabilization.	Chowdhury et al. (2023)

Membrane compositions are reported as molar ratios in the corresponding primary studies. CO-raft, cholesterol-rich raft; GM-raft, GM1-enriched raft; PS-raft, phosphatidylserine-enriched raft; CG, coarse-grained; AA, all-atom; PHF, paired helical filament; SF, straight filament; PHF6, paired helical filament 6 motif; CHOL, cholesterol; DLPC, dilinoleoylphosphatidylcholine; DPPC, dipalmitoylphosphatidylcholine; DOPC, dioleoylphosphatidylcholine; DOPS, dioleoylphosphatidylserine; POPC, palmitoyl-oleoyl-phosphatidylcholine; POPE, palmitoyl-oleoyl-phosphatidylethanolamine; POPS, palmitoyl-oleoyl-phosphatidylserine; PSM, palmitoylsphingomyelin. Lipid compositions: CO-raft, DPPC:DLPC:CHOL = 0.42:0.28:0.30; GM-raft, GM1:DPPC:DLPC:CHOL = 0.02:0.43:0.30:0.25; PS-raft, POPS:DPPC:DLPC:CHOL = 0.08:0.34:0.28:0.30; neuronal membrane model, POPC:CHOL:GM1:POPS:POPE:PSM = 38:20:4:5:24:9.

Most available Tau–membrane simulations examine non-modified fragments or Alzheimer’s disease-derived fibrillar states rather than PD-specific phosphoconformers. Their relevance to PD is therefore mechanistic and hypothesis-generating, and future studies should incorporate Tau states reproducibly identified in PD-derived samples.

### 6.4 Chemical microstates and disease-specific filament contexts

Chemical microstates and pre-existing filament architecture provide additional context for Tau structural remodeling. Alternative neutral histidine tautomers altered β-sheet propensity and intramolecular contacts in an isolated R3 fragment (Li et al., 2021), while Alzheimer’s disease and Pick’s disease filaments exhibit distinct fibrillar folds (Fitzpatrick et al., 2017; Falcon et al., 2018; Shi et al., 2021). In a narrow Pick filament model, phosphorylation and repeat-specific substitutions produced distinct local electrostatic and PGGG-motif responses in R1 and R3 (Sahayaraj et al., 2023). These findings emphasize that PTM effects depend on local sequence and pre-existing structural context and should not be generalized across disease states. PD-focused modeling should therefore prioritize Tau isoforms and PTM patterns supported by PD-derived evidence.

## 7 In silico and bioinformatics-based strategies for Tau-targeted drug discovery

The translational value of Tau-directed computational drug discovery in PD depends on selecting molecular states supported by phosphoproteomic, biochemical, or pathological evidence. Emerging therapeutic approaches include modulation of Tau phosphorylation, targeted degradation through Tau-directed protein degraders, directed dephosphorylation through PhosTACs, and direct targeting of aggregation-prone or fibrillar conformations. Within this context, computational methods can support conformational-ensemble modeling, dynamic pocket detection, molecular docking, and molecular dynamics refinement. These approaches should be applied to defined Tau isoforms, post-translational modification patterns, and aggregation states rather than to Tau as an interchangeable molecular target (Silva et al., 2022; Hu et al., 2023; Seidler et al., 2022). Tau-targeted degradation has been demonstrated in patient-derived neuronal models of frontotemporal tauopathy, whereas Tau-directed PhosTACs have induced PP2A-dependent dephosphorylation in engineered cellular systems; neither approach has yet established therapeutic efficacy in PD.

Tau lowering has also reached clinical evaluation through antisense oligonucleotides. In mild Alzheimer’s disease, the Tau-targeting antisense oligonucleotide MAPTRx/BIIB080 (diranersen) reduced CSF Tau concentrations in a phase 1b trial (Mummery et al., 2023). It has subsequently been evaluated in the phase 2 CELIA study (Biogen, 2026). These clinical data derive from Alzheimer’s disease and do not establish therapeutic efficacy in PD.

Conventional molecular docking is challenging for soluble full-length Tau because this intrinsically disordered protein exists as an ensemble of rapidly interconverting conformations rather than as a single folded receptor. No persistent, experimentally validated small-molecule pocket has been established for physiological soluble Tau. Therefore, Tau-targeted virtual screening should first define the molecular state under investigation, including the isoform, post-translational modification pattern, aggregation state, and molecular environment. Soluble non-modified Tau, phosphorylated Tau, microtubule-bound fragments, oligomers, and fibrils should not be treated as interchangeable docking receptors (Mukrasch et al., 2009).

For soluble and phosphorylated Tau, these limitations can be partially addressed using experimentally informed conformational models, molecular dynamics simulations, clustering, dynamic pocket mapping, and ensemble docking. The PHF6* (^275VQIINK^280) and PHF6 (^306VQIVYK^311) motifs constitute structurally characterized aggregation hotspots that may support inhibitor design, although they represent amyloid-forming interfaces rather than conventional permanent pockets (von Bergen et al., 2000; Li and Lee, 2006). A model-based docking study of phosphorylated Tau also proposed a ligand-accessible region within approximately residues 267–312, including Ser285, Ser289, Ser293, Tyr310, and neighboring residues (Pradeepkiran and Reddy, 2019). Because this region was identified using a homology-modeled partial Tau construct, it should be regarded as a conformer-dependent hypothesis rather than an experimentally validated persistent pocket in full-length phosphorylated Tau.

More clearly defined ligand-accessible sites have been identified in ordered fibrillar Tau. In Alzheimer’s disease-derived paired helical filaments, Site 1 is a polar inter-protofilament cleft within the R3–R4 region of the microtubule-binding repeat domain, bordered by Asn327, His329, Glu338, and Lys340. This cleft supported structure-based docking and experimental identification of fibril-disrupting compounds. However, the site is specific to the ordered AD fibrillar architecture and should not be assumed to exist in soluble, differently modified, or PD-associated Tau conformations. Tau-directed docking should therefore be interpreted as a state-specific strategy and combined with molecular dynamics refinement and experimental validation (Seidler et al., 2022).

### 7.1 Natural products and structure-based discovery

Natural products have been investigated as potential modulators of Tau aggregation and fibril stability. Baicalein inhibited heparin-induced fibrillization of full-length Tau and redirected the protein toward oligomeric species reported to have reduced toxicity. Curcumin suppressed aggregation of 1N4R Tau and a modified PHF6-derived peptide, whereas rosmarinic acid reduced Tau fibrillization and β-sheet-associated structural signals. EGCG has also been visualized within ligand-accessible clefts of AD brain-derived Tau filaments and shown to initiate destabilization. These compounds provide mechanistic probes and potential starting scaffolds, but their selectivity, brain exposure, bioavailability, and pharmacokinetic properties require compound-specific evaluation (Sonawane et al., 2021; Bijari et al., 2018; Cornejo et al., 2017; Seidler et al., 2022).

Yin et al. combined biochemical assays, molecular dynamics simulations, and cellular evaluation to examine grape seed proanthocyanidins (GSPs). GSPs inhibited Tau repeat-domain aggregation, reduced β-sheet formation, disassembled pre-formed Tau-RD fibrils, and attenuated the cytotoxicity associated with Tau-RD aggregates. The simulations indicated that hydrogen bonding, hydrophobic contacts, and π–π stacking contributed to GSP–Tau-RD association. These findings support GSPs as mechanistic anti-aggregation probes, but they were obtained using an AD-oriented Tau-RD system and do not establish efficacy, target engagement, or pharmacological activity in PD (Yin et al., 2023).

Seidler et al. (2022) resolved EGCG within the R3–R4 Site 1 cleft of AD-derived paired helical filaments and used this structure for virtual screening. Four CNS-Set compounds showed IC_50_ values below 5 μM in a Tau-seeding assay, and CNS-11 promoted fibril disassembly but displayed cytotoxicity. MD simulations indicated that EGCG and CNS-11 perturb interlayer contacts, with EGCG increasing local Tau-layer separation and disrupting the hydrogen-bond network. These results support a mechanism in which ligand binding weakens interlayer stabilization and promotes solvent-accessible fibril disruption, linking a resolved fibrillar pharmacophore to docking, MD, and experimental validation.

Together, these studies show how experimentally resolved fibrillar structures can support pharmacophore definition, virtual screening, molecular dynamics refinement, and biochemical validation. The most structurally developed examples, however, derive from Alzheimer’s disease filament architectures and are therefore mechanistic rather than PD-specific. Candidate compounds or docking sites should be advanced only after confirming that the targeted Tau assembly, phosphosignature, or conformational state is present in PD-relevant tissue or neuronal models.

### 7.2 Ligand-based and chemoinformatic approaches

Ligand-based design complements structure-based approaches by identifying recurring molecular features among compounds tested for Tau anti-aggregation activity. Pinzi et al. (2021) curated 49,284 unique Tau ligands from ChEMBL using thioflavin T or thioflavin S fluorescence assays against human Tau. These entries should not be interpreted as equally potent or experimentally equivalent inhibitors. For binary similarity analyses, 905 compounds with reported potency below 500 nM were assigned to the active set, whereas 47,246 compounds above 1 μM formed the inactive comparison set. The analysis therefore compared defined activity groups rather than treating the complete dataset as a uniform collection of Tau inhibitors.

Among the more active ligands, recurrent fragment combinations suggested a general framework comprising two aromatic or heteroaromatic rings connected by a two- to seven-atom linker, frequently containing hydrogen-bond acceptors or unsaturated functional groups. Pinzi et al. identified related features in xalsalate and curcumin and in the natural compounds xanthohumol and licochalcone A. These examples connect the ChEMBL-derived signatures with previously investigated Tau aggregation modulators. However, this comparison is structural and chemoinformatic rather than a direct ranking of pharmacological potency because the compounds were evaluated using different assay conditions, activity endpoints, and stages of development. Methylene blue can additionally serve as a benchmark compound in Tau fibrillization assays (Hattori et al., 2008; Soeda et al., 2019). It was not the comparator used by Pinzi et al. (2021), and its effects depend on assay format and the Tau assembly state.

Although molecular docking was not performed in this study, the chemoinformatic signatures identified by Pinzi et al. can guide pharmacophore development and similarity-based screening for later state-specific docking and molecular dynamics refinement. Such prioritization does not demonstrate binding to a defined Tau conformer or therapeutic efficacy in PD. Candidate ligands should therefore advance only after the relevant Tau isoform, post-translational modification pattern, and aggregation state have been experimentally supported.

## 8 Artificial intelligence and network medicine in Tau-targeted therapeutic discovery

Tau biology presents substantial challenges for conventional drug discovery because of its six major adult-brain isoforms, extensive post-translational modification landscape, dynamic conformational states, and interactions with other neurodegeneration-related proteins (Goedert et al., 1989a; Wang and Mandelkow, 2016). Tau should therefore be considered both a state-dependent molecular target and a component of interconnected cellular pathways.

Artificial intelligence and network-medicine approaches can complement structure- and ligand-based methods by integrating molecular, pharmacological, and omics data (Hopkins, 2008; Sadegh et al., 2021; Das et al., 2024). They can identify disease-associated network modules, prioritize drug-repurposing candidates, and screen representative Tau conformations. Most Tau-related applications, however, remain computational or derive from Alzheimer’s disease and other non-PD contexts. Their relevance to PD therefore requires validation in PD-derived samples and disease-relevant neuronal models.

### 8.1 Network medicine: positioning Tau-related mechanisms within disease interactomes

Network medicine places Tau-related mechanisms within protein-interaction and regulatory networks and can prioritize repurposing candidates according to their relationship with disease-associated modules (Hopkins, 2008; Sadegh et al., 2021). Complementary methods include mechanism-of-action mapping, diffusion-based algorithms, and transcriptomic signature reversal, including Gene Set Enrichment Analysis (Sadegh et al., 2021; Subramanian et al., 2005). These approaches generate prioritization hypotheses but do not independently establish efficacy, target engagement, or safety.

Funari et al. (2025) provide an Alzheimer’s disease-based methodological example combining network drug repositioning, predicted cardiac-safety filtering, and transcriptomic signature reversal. Their workflow narrowed 179 initial candidates to 133 after network-based safety filtering, with 25 receiving additional transcriptomic support; none was experimentally validated. This framework is transferable rather than Tau- or PD-specific. Applied to PD, similar analyses could integrate Tau phosphosignatures with α-synuclein pathways and candidate targets, followed by validation in PD-relevant neuronal models.

### 8.2 AI-powered virtual screening and *de novo* molecular design

Artificial intelligence and machine-learning methods are increasingly being investigated for Tau-targeting compound discovery. Das et al. used the AI-assisted ligand-based platform PyRMD to screen approximately 12 million ZINC compounds. After activity prediction, filtering, and diversity selection, 2,367 compounds were docked against ten Tau conformations generated by replica-exchange molecular dynamics. Subsequent in silico ADME, molecular dynamics, and MM/PBSA analyses prioritized seven potential Tau aggregation inhibitors. Because these candidates were identified exclusively computationally, biochemical, biophysical, and cellular validation remains necessary. This workflow is therefore best described as AI-assisted virtual screening rather than experimentally validated drug discovery (Das et al., 2024).

Generative approaches extend this strategy by creating new molecular structures rather than selecting compounds exclusively from existing libraries. Reinforcement-learning methods can guide molecular generators toward predefined objectives, including predicted biological activity, physicochemical properties, or similarity to a reference structure (Olivecrona et al., 2017). Variational autoencoders provide a complementary strategy by mapping discrete molecular representations into a continuous latent space that can be explored for property optimization and molecular generation (Gómez-Bombarelli et al., 2018). These approaches provide a methodological basis for scaffold generation and multi-objective molecular design, but their outputs remain dependent on the quality of the training data, molecular representation, predictive models, and optimization functions.

Prospective non-Tau studies show that reinforcement learning can be paired with structure-based design and experimental testing. Thomas et al. (2025) used a chemical language model and reinforcement learning to design adenosine A₂A receptor ligands; nine compounds were synthesized, eight showed measurable binding, and several displayed functional activity, including three nanomolar ligands. Equivalent prospective validation remains limited for Tau. Tau applications will require a defined isoform, PTM pattern, and aggregation state, together with optimization for blood–brain barrier penetration, synthetic accessibility, selectivity, and pharmacokinetic properties. Generated candidates should then undergo ensemble docking, molecular dynamics refinement, biochemical binding or aggregation assays, cellular target-engagement studies, and toxicity assessment. Generative AI therefore complements rather than replaces experimentally informed structural modeling and biological validation.

For application to PD, future computational workflows should incorporate experimentally supported Tau states derived from PD tissues, biofluids, or disease-relevant neuronal models. Where sufficient data are available, model development and candidate prioritization should also consider reproducible Tau phosphosignatures, α-synuclein-associated mechanisms, relevant genetic backgrounds, and dopaminergic neuronal vulnerability. Current predictions derived predominantly from Alzheimer’s disease, general tauopathy datasets, or non-disease-specific Tau models should therefore be regarded as transferable hypotheses rather than evidence of activity against PD-specific Tau conformers. Their relevance will require validation in PD-derived samples and neuronal systems that reproduce the targeted molecular state.

### 8.3 AI-based prediction of Tau aggregation-related features and PTM effects

Machine-learning methods can support prediction at different biological and structural scales. General sequence-based tools such as AmyloGram identify amyloidogenic peptide segments but are not Tau-specific and do not capture full-length Tau heterogeneity, combinations of PTMs, or disease-specific fibril architectures (Burdukiewicz et al., 2017). Tau-specific approaches provide more direct structural information: Mullapudi et al. used in silico alanine scanning and random-forest classification across 27 Tau fibril polymorphs to identify interaction hotspots, with selected predictions evaluated *in vitro* and in cellular aggregation assays (Mullapudi et al., 2023). However, this approach classified resolved fibrillar architectures rather than soluble full-length Tau behavior, neuronal toxicity, or clinical progression.

Phosphorylation-sensitive interactions can also be prioritized computationally. DeepPhosPPI uses protein-language-model embeddings and neural-network architectures to identify potentially functional phosphorylation sites and predict their effects on protein–protein interactions, but Tau-specific performance and PD specificity remain unestablished (Gong et al., 2025). At a broader disease level, Venkatesh et al. integrated genomic, transcriptomic, and proteomic association data in Alzheimer’s disease; this study supports the potential of multi-omics integration but did not model combinatorial Tau PTMs or Tau–α-synuclein-dependent toxicity (Venkatesh et al., 2025). Together, these approaches can prioritize amyloidogenic features, fibril-stabilizing interactions, and phosphorylation-sensitive networks, but future PD models should incorporate reproducible Tau PTM patterns and undergo prospective biochemical, structural, and cellular validation.

## 9 Discussion

The evidence reviewed here supports Tau, particularly its dysregulated phosphorylation and interactions with α-synuclein, as a context-dependent contributor to Parkinson’s disease rather than a uniform or independently sufficient driver. Genetic association at the MAPT locus, Tau-related alterations in post-mortem PD brain, and experimental Tau–α-synuclein cross-talk indicate that abnormal Tau may increase neuronal vulnerability in a subset of patients (Simón-Sánchez et al., 2009; Wills et al., 2010; Padilla-Godínez et al., 2025). Its clinical relevance likely varies with genetic background, α-synuclein pathology, brain region, disease stage, and Tau molecular state. A central question is whether Tau acts as a driver in defined PD subgroups, a modifier of α-synuclein-associated pathology, or a marker of concomitant neurodegeneration.

Direct phosphosite-resolved data from human PD brain remain limited, and validated circulating Tau phosphosignatures for diagnosis, stratification, or progression monitoring have not been established. Patient-derived neuronal models bridge genetic susceptibility and molecular mechanisms, but findings from individual LRRK2- or GBA-associated systems cannot be generalized to all PD. Likewise, much structural and molecular-dynamics evidence derives from Alzheimer’s disease, other tauopathies, recombinant fragments, or experimental fibrils rather than Tau states identified directly in PD. These systems provide valuable mechanistic information but should be treated as transferable hypotheses rather than demonstrations of PD-specific Tau mechanisms (Bogetofte et al., 2023; Fitzpatrick et al., 2017; Falcon et al., 2018; Seidler et al., 2022).

Tau’s intrinsically disordered and post-translationally heterogeneous nature also limits conventional structure-based targeting. Phosphorylation is not uniformly disruptive, and its effects depend on residue position, stoichiometry, combinations of phosphosites, and molecular environment. Molecular dynamics can resolve some of these context-dependent effects, but outcomes depend on the molecular construct, starting state, force field, and sampling strategy. Similarly, ensemble docking, chemoinformatics, artificial intelligence, and network-medicine approaches can prioritize state-specific hypotheses, but they do not establish disease specificity or therapeutic efficacy. Experimental degraders, PhosTACs, fibril-disrupting compounds, and AI-assisted screening demonstrate technical feasibility (Silva et al., 2022; Hu et al., 2023; Seidler et al., 2022; Das et al., 2024); candidates still require biochemical confirmation, selectivity and toxicity assessment, and functional testing in PD-relevant models.

Progress will require reproducible mapping of Tau phosphosites in well-characterized PD tissues, biofluids, and patient-derived neuronal systems, ideally across longitudinal and genetically diverse cohorts. Candidate phosphosignatures should be evaluated alongside α-synuclein pathology to identify biologically distinct PD subgroups rather than nonspecific neurodegeneration or concomitant Alzheimer-type pathology. Structural studies should increasingly model Tau isoforms and PTM patterns observed in PD, and therapeutic mechanisms should follow a staged validation path from phosphoproteomic discovery through structural modeling, biochemical or biophysical testing, cellular target engagement, and PD-relevant patient-derived or *in vivo* models.

Integration with genomics, transcriptomics, proteomics, metabolomics, and lipidomics may further define molecular subgroups, while advances in structural biology and artificial intelligence may improve state-specific therapeutic prioritization. These developments could support biomarker-informed patient stratification and mechanism-guided testing, but personalized Tau-directed intervention in PD remains a prospective objective requiring validation across independent cohorts. The central opportunity is not to redefine PD as a primary tauopathy, but to determine when, where, and in which patients Tau dysregulation contributes meaningfully to disease and can be targeted with measurable clinical benefit.
